# Performance Optimization of a High-Speed Permanent Magnet Synchronous Motor Drive System for Formula Electric Vehicle Application

**DOI:** 10.3390/s25103156

**Published:** 2025-05-16

**Authors:** Mahmoud Ibrahim, Oskar Järg, Raigo Seppago, Anton Rassõlkin

**Affiliations:** 1 Department of Electrical Power Engineering and Mechatronics, School of Engineering, Tallinn University of Technology, 19086 Tallinn, Estonia; mahmoh@taltech.ee; 2 Department of Mechanical and Industrial Engineering, School of Engineering, Tallinn University of Technology, 19086 Tallinn, Estonia; oskar.jarg@taltech.ee; 3 Department of Computer Systems, School of Information Technologies, Tallinn University of Technology, 19086 Tallinn, Estonia; rasepp@taltech.ee

**Keywords:** permanent magnet synchronous motor, MTPA, vector control, ZDAC

## Abstract

The proliferation of electric vehicle (EV) racing competitions, such as Formula electric vehicle (FEV) competitions, has intensified the quest for high-performance electric propulsion systems. High-speed permanent magnet synchronous motors (PMSMs) for FEVs necessitate an optimized control strategy that adeptly manages the complex interplay between electromagnetic torque production and minimal power loss, ensuring peak operational efficiency and performance stability across the full speed range. This paper delves into the optimization of high-speed PMSM, pivotal for its application in FEVs. It begins with a thorough overview of the FEV motor’s basic principles, followed by the derivation of a detailed mathematical model that lays the groundwork for subsequent analyses. Utilizing MATLAB/Simulink, a simulation model of the motor drive system was constructed. The proposed strategy synergizes the principles of maximum torque per ampere (MTPA) with the flux weakening control technique instead of conventional zero direct axis current (ZDAC), aiming to push the boundaries of motor performance while navigating the inherent limitations of high-speed operation. Covariance matrix adaptation evolution strategy (CMA-ES) was deployed to determine the optimal d-q axis current ratio achieving maximum operating torque without overdesign problems. The implementation of the optimized control strategy was rigorously tested on the simulation model, with subsequent validation conducted on a real test bench setup. The outcomes of the proposed technique reveal that the tailored control strategy significantly elevates motor torque performance by almost 22%, marking a pivotal advancement in the domain of high-speed PMSM.

## 1. Introduction

In recent years, the landscape of motorsports has witnessed a notable shift with the burgeoning popularity of FEV racing. As a pivotal player in the forefront of EV racing, the FEV stands poised to redefine the future of motorsports, heralding a paradigm shift that anticipates the eventual replacement of conventional Formula racing vehicles [1].

At the heart of the FEV lies its powertrain, and notably, the electric motor stands as the core element defining its performance. Unlike conventional EVs, FEV motors are engineered to meet the unique demands of high-speed racing circuits. These motors exhibit exceptional specifications, characterized by a delicate balance between power density, torque response, and speed range. Low-inertia and high-speed PMSMs have become increasingly prevalent in FEV applications, owing to their superior efficiency, compactness, and robust performance under the high-load conditions typical of racing environments [2]. PMSMs are distinguished by their ability to provide a high torque-to-weight ratio, a critical factor in enhancing the acceleration and agility of Formula EVs on track [3]. Despite the notable advantages of high-speed PMSMs in FEVs, several challenges persist in their application. One critical issue is the difficulty in maintaining optimal torque output and efficiency across varying speed ranges and load conditions, which can lead to suboptimal performance and increased energy consumption. Another problem is the thermal management of the motor, as high-speed operations can result in significant heat generation, potentially compromising motor longevity and reliability. Additionally, the dynamic nature of racing conditions demands rapid and precise control responses, which current control strategies may not adequately provide [4].

Recent studies have explored various aspects of PMSM application in Formula EVs, including thermal management, control strategies, and performance optimization. The advancements in vector control strategies for high-speed PMSMs in FEVs emphasize optimization and efficiency. Elsonbaty et al. [5] proposed an efficient vector control strategy specifically designed for EV applications, focusing on achieving high-speed operation through simplified control strategies under specific constraints. Wei et al. [6] analyzed the dynamic torque response of high-speed control strategies in PMSMs, providing insights into the application of these motors in EV drives that are not commonly utilized in real-world scenarios. Zhao et al. [7] further explored the potential of sliding mode vector control systems in enhancing the torque and speed control of PMSMs at various operational stages, thus promoting more stable and efficient motor performance. Murali et al. [8] assessed the performance of PMSMs under different control strategies, highlighting the advantages of finite control set model predictive control (FCS-MPC) over traditional field-oriented control (FOC). Their findings suggested that FCS-MPC provides superior dynamic and steady-state responses, which are crucial for the varying speed and torque requirements of FEV. Li and Song [9] explored the impact of magnetic circuit saturation on the performance of PMSMs, proposing a nonlinear model-based control strategy to enhance motor efficiency and adaptability under different operating conditions. Their research highlighted the complexities of PMSM control in EVs and the need for sophisticated control algorithms to achieve optimal performance, particularly in the high-stakes environment of FEV racing. In the context of Formula Student racing, Da Silva and Waltrich [10] explored the development of a field-oriented control (FOC) voltage source inverter with space vector modulation (SVM) for enhanced motor control. Their research addressed the importance of tailored control solutions in competitive racing environments, where precise torque and speed control can significantly impact performance. Jujjuvarapu and Rajulapati [11] employed machine learning to refine MTPA implementation for interior PMSMs, introducing an adaptive mechanism that significantly improves torque accuracy and energy efficiency in EV drives. Their framework notably reduced energy losses under varying load conditions, a particularly relevant factor for competitive applications such as Formula EV. Similarly, Wu et al. [12] proposed a robust, self-tuning MTPA control algorithm designed for PMSM-based EVs, enhancing adaptability to dynamic driving conditions. The algorithm’s ability to maintain optimal performance under real-time variation represents a substantial step forward in reliable high-speed EV operation. Shen et al. [13] conducted a comparative study on PMSM efficiency optimization techniques, contrasting MTPA with loss minimization control (LMC). Their findings underscore MTPA’s superiority in dynamic efficiency, reinforcing its status as the preferred approach in speed-variable applications like racing. Building on the theme of control stability at high speeds, Arias et al. [4] presented a comprehensive analysis of torque control stability issues in surface-mounted PMSMs. Their study introduced a discrete-time modeling framework and a novel speed-adaptive control strategy that extends operational speed ranges while mitigating the limitations of conventional FOC methods—an innovation especially relevant for high-speed FEV applications. Dianov and Anuchin [14] developed an adaptive MTPA algorithm suited to sensorless control environments, which enhances robustness under harsh real-world dynamics such as rapid throttle changes or regenerative braking during sharp corners, typical in FEVs. Mondal et al. [15] proposed a novel FOC-based MTPA enhancement specifically tailored for electric traction motors in racing, showing improved energy efficiency without compromising torque response even during limit-cycle operations. In addition, Yang and Lu [16] introduced a model-free predictive MTPA controller that directly calculates the optimal current vector, eliminating the dependency on real-time motor parameters—making it ideal for embedded systems in competitive FEVs. While notable progress has been made in advancing control strategies for high-speed PMSMs in FEVs, key challenges persist that existing approaches have not been fully addressed. Despite improvements in vector control, sliding mode control, and model predictive control, current strategies often fall short in simultaneously optimizing torque output and maintaining efficiency across the full speed range. Most existing research has tackled these issues in isolation, leaving a critical gap in the development of a unified control strategy that can robustly manage the complex demands of real-world racing conditions. Furthermore, many of the proposed methods are tested only in simulated environments and lack thorough validation under the intense and unpredictable conditions of competitive racing.

**Figure 1:**
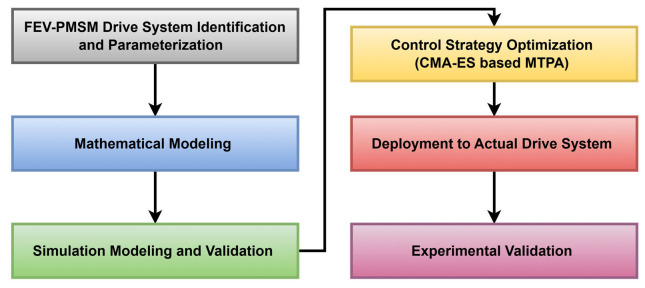
FEV-PMSM performance optimization progression workflow.

The proposed study bridges the gap by incorporating an optimal control strategy tailored for a high-speed PMSM drive used in FEV applications. The research aims to demonstrate significant improvements in FEV motor performance through a comprehensive approach that includes mathematical modeling, simulation, and validation through lab-based testbench experiments. Figure 1 shows a workflow diagram of the proposed methodology for the FEV-PMSM performance optimization.

This paper is organized as follows. Section 1 introduces an overview of the recent advancements in high-speed PMSM for FEV applications. Section 2 proposes a complete system description, including high-speed PMSM drive characteristics and FEV application requirements. Section 3 addresses the drive system’s modeling procedures. Section 4 proposes the development and simulation analysis of the motor optimal control strategy. In Section 5, experimental validation is highlighted alongside the research outcomes. Finally, the paper’s conclusions are presented in Section 6.

## 2. Formula EV PMSM Basic Principles

### 2.1. FEV PMSM Ideal Torque Speed Profile

**Figure 2:**
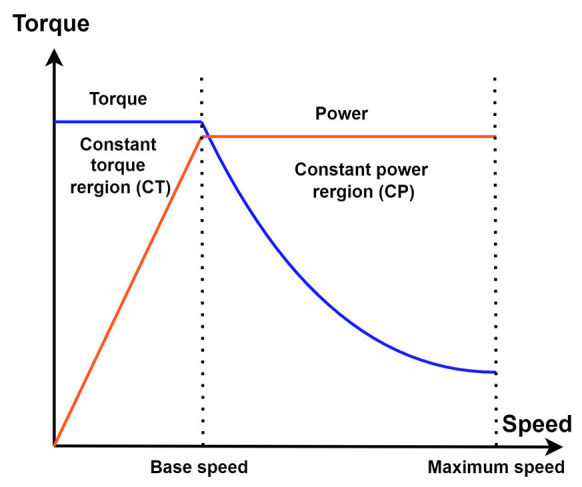
Ideal torque-power/speed profile of EV.

For EVs, the torque-speed profile is a critical determinant in reaching ultimate vehicle performance, shaping factors such as efficiency, driving range, and the overall driving experience. EVs are specifically engineered with a torque profile that delivers high torque at low speeds to facilitate brisk urban acceleration. This high initial torque is crucial for enabling quick starts and efficient, short-distance urban travel. Generally, the torque profile in EVs can be divided into two main regions: the constant torque (CT) region and the constant power (CP) region, as shown in Figure 2. In the CT region, the torque reaches its peak early on and is maintained consistently until a certain speed threshold is known as base speed. This region is vital in providing the necessary acceleration from a standstill, enhancing the vehicle’s responsiveness. As the vehicle’s speed increases beyond this threshold, it transitions into the CP region, where the torque starts to decline inversely with speed. This design ensures that the power remains constant, optimizing the motor’s efficiency at higher speeds. This bipartite division of the torque-speed profile into CT and CP regions is intentional and serves multiple purposes. The constant torque region allows for significant acceleration without a high-power demand, which helps preserve battery life and improves the range. On the other hand, the constant power region optimizes high-speed travel efficiency and prevents the motor from operating beyond its capacity, which can lead to increased wear and energy inefficiency. By configuring the torque to decrease as speed increases in this region, the vehicle conserves energy, thereby extending the driving range per charge. These strategic design choices in shaping the torque-speed profile ensure that EVs maintain a balance between robust performance and practical energy utilization.

### 2.2. FEV PMSM Drive System

The PMSM drive system used in the study is the Formula Student Tallinn FEV. The vehicle power train adopted a hub motor configuration, which allows for unparalleled responsiveness and torque delivery, as the power from the motors is transmitted directly to the wheels without the need for complex transmission systems. Such an arrangement significantly reduces powertrain losses, ensuring that the maximum amount of power generated by the motors is effectively used for propulsion. The motor is driven by a traction inverter utilizing silicon carbide transistor (SiC) technology. Table 1 highlights the FEV motor parameters.

## 3. FEV Motor Drive System Modeling and Simulation

### 3.1. PMSM Mathematical Model

The mathematical modeling of a high-speed PMSM drive requires a detailed understanding of both the electrical and thermal characteristics. This subsection provides the mathematical framework for the PMSM drive, incorporating thermal effects. Thermal considerations are crucial in the mathematical modeling of high-speed PMSM drives, especially for applications like FEV, where the motors are subject to extreme operating conditions. The following equations represent the mathematical model of the PMSM [17].

Stator d-q voltage equations can be developed as follows:

(1) $$V_{d}=R_{s}I_{d}+\frac{d}{dt}\lambda_{d}{+E}_{d}$$

(2) $${\;V}_{q}=R_{s}I_{q}+\frac{d}{dt}\lambda_{q}+E_{q},$$

where $R_{s\;}$ is the stator resistance, *I_d_* and *I_q_* denote the corresponding stator current components. The variables $\lambda_{d}$ and $\lambda_{q}$ refer to the stator flux linkages in the d- and q-axes, The components $E_{d}\;$ and $E_{q}$, the back electromotive forces (EMFs), are associated with the d- and q-axes, respectively, arising due to the interaction between the rotor’s magnetic field and rotational motion.

The flux linkage equation can be given as below:

(3) $${\;\lambda }_{d}=\lambda_{pm}+L_{d}I_{d}$$

(4) $$\lambda_{q}=L_{q}I_{q},$$

The motor temperature affects its electrical and mechanical characteristics. Incorporating temperature effects requires additional equations and parameters related to temperature.

Resistance variation due to temperature is calculated as:

(5) $$R_{s}=R_{s0}\cdot \left(1+\alpha T\cdot \left(T-T_{0}\right)\right)$$

where $R_{s0}$ is the resistance at the reference temperature (*T*_0_), and *αT* is the temperature coefficient of resistance. Then, the stator voltage equations can be rewritten as follows:

d-axis voltage equation:

(6) $$V_{d}=(R_{s0}\cdot \left(1+\alpha T\cdot \left(T-T_{0}\right)\right))\cdot I_{d}+L_{s}\cdot \frac{dI_{d}}{dt}-\omega_{r}\left(I_{q}\right)\;$$

q-axis voltage equation:

(7) $$V_{q}=(R_{s0}\cdot (1+\alpha T\cdot (T-T_{0})))\cdot I_{q}+L_{\;s}\cdot dI_{q}/dt-\omega_{r}\cdot (I_{d})$$

(8) $$T_{e}=\frac{3}{2}\cdot p\cdot (\lambda d\cdot I_{q}-\lambda q\cdot I_{d})$$

(9) $$J\cdot \frac{d\omega r}{dt}=T_{e}-T_{l}-B\cdot \omega_{r}$$

The motor drive system utilizes the FOC strategy with flux weakening capability, which is used to extend the operating speed range of the motor beyond its base speed. The concept of FOC with flux weakening is based on reducing the magnetic flux in the stator to limit the EMF and stay within the voltage limits of the inverter. The strategy comprises Park transformer, current PI controller, inverse Park transformer, and a flux weakening controller, which adjusts the d-axis current reference $I_{d}^{*}$ to reduce the magnetic flux in the stator when the motor speed exceeds the base speed. The flux weakening current reference $I_{dfw}^{*}$ is determined based on the motor speed ωr and the available voltage margin as follows:

(10) $$I_{dfw}^{*}=\left\{\begin{array}{c} 0\;\omega_{r}\leq \omega_{b}\;\\ -K_{fw}\left(\omega_{r}-\omega_{b}\right)\omega_{r}>\omega_{b} \end{array}\right.$$

**Figure 3:**
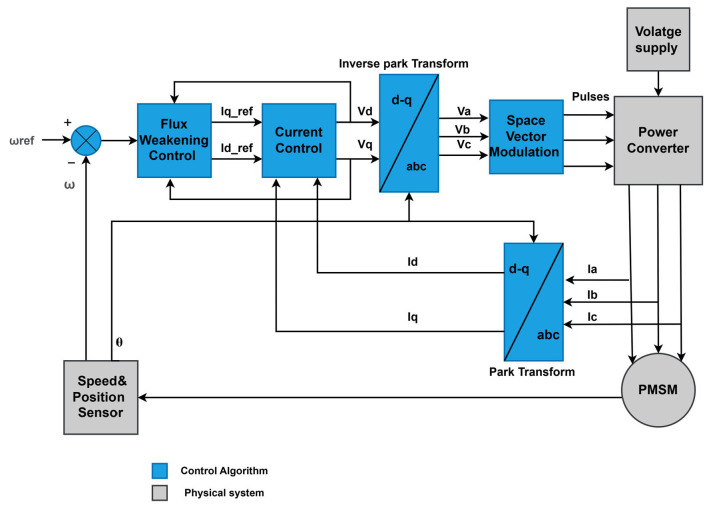
PMSM FOC control strategy diagram.

Figure 3 shows the control scheme of the FEV PMSM drive system.

### 3.2. FEV Motor Drive System Simulation Model

A physics-based simulation model of the motor drive system was developed using MATLAB/Simulink 2024a. The model comprises four subsystems replicating the motor drive system components.

The first subsystem includes setting points, which are the testing conditions of the motor drive, including the DC link voltage, the load torque, the motor, and inverter temperatures. These parameters are crucial in defining the operating environment of the motor drive system and ensuring the simulation accurately reflects real-world conditions. The second subsystem represents the control strategy defined in the previous subsection. This included implementing the Clarke and Park transformations, current controllers, and flux weakening controllers. The control algorithm was modeled using Simulink blocks and mathematical functions. The third subsystem represents the physical components, including the PMSM model and the inverter model. The PMSM model was constructed using the fundamental equations governing the motor’s electrical and mechanical dynamics. It included the interaction between the stator and rotor magnetic fields, torque production, and speed dynamics. The inverter model block was built using the PLECS blockset, which provides a detailed representation of the power electronic switches, their switching behavior, and the associated control logic. This allowed for accurate simulation of the inverter’s operation, including its response to the control signals and its interaction with the motor. Additionally, the PLECS blockset enabled the inclusion of detailed thermal modeling of the inverter components, providing insights into the thermal behavior and cooling requirements of the power electronics. This comprehensive approach ensured that both electrical and thermal aspects of the motor drive system were considered, leading to a more accurate and reliable simulation model.

**Figure 4:**
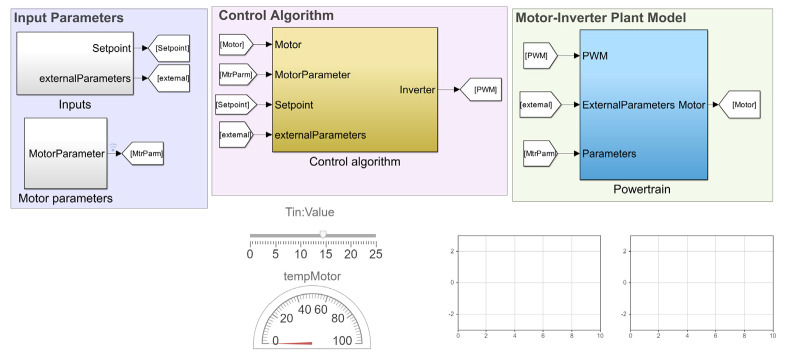
FEV motor drive simulation model.

The integration of these subsystems into a unified Simulink model allowed for thorough analysis and testing of the motor drive system. Consequently, the simulation model was constructed as torque-based, with the input being the reference torque. This approach mirrors practical applications where the motor control system primarily operates based on torque commands, translating the driver’s input into precise torque requests for optimal handling and performance. Figure 4 shows the proposed simulation model of the FEV drive system.

## 4. FEV Motor Drive System Performance Optimization

### 4.1. Control Strategy Optimization

Operating the motor in the constant torque (CT) region at a higher torque level is crucial in enhancing the overall performance of the FEV, especially during drifts on racing tracks. In these scenarios, maintaining high torque is essential to achieve quick acceleration and precise control, which are vital for competitive performance. Higher torque in the CT region improves the vehicle’s responsiveness and agility, allowing drivers to navigate sharp turns and sudden maneuvers more effectively.

To optimize the control strategy for this purpose, the maximum torque per amper (MTPA) technique was applied instead of the zero direct-axis current (ZDAC) control. The MTPA technique adjusts the current vector to maximize the torque output for a given current level, thus allowing the motor to produce more torque in the CT region compared to the ZDAC approach. This enhancement results in better performance during critical phases of the race, such as cornering and overtaking.

The idea is based on achieving additional reluctance torque added to the overall motor torque produced by the permanent magnet, using the saliency of the motor because it is an interior PMSM. The interior PMSM has distinct d-axis and q-axis inductance (Ld, Lq), which can be leveraged to generate reluctance torque. By appropriately controlling the d-axis current (Id), the MTPA technique exploits the motor’s magnetic saliency to enhance the overall torque output.

In practical terms, the MTPA strategy calculates the optimal d-axis current (Id) to be applied along with the q-axis current (Iq), ensuring that the combination of magnetic torque from the permanent magnets and reluctance torque from the rotor’s saliency is maximized. This results in higher efficiency and better utilization of the motor capabilities, especially in the CT region where torque demands are high.

Furthermore, while implementing MTPA increases the torque in the CT region, it does have implications for the motor’s base speed and the extent of the CP region. Specifically, the base speed decreases slightly, leading to a wider CP region. This trade-off is managed carefully to ensure optimal torque across the motor’s entire operating range without leading to overdesign issues, such as excessive thermal loads or mechanical stress.

### 4.2. Covariance Matrix Adaptation Evolution Strategy (CMA-ES)

To achieve the optimal relationship between the d-axis and q-axis currents for the MTPA strategy, the Covariance Matrix Adaptation Evolution Strategy (CMA-ES) was employed. CMA-ES is an advanced algorithm known for its robustness and efficiency in handling high-dimensional, nonlinear, and complex optimization problems. The goal was to find the optimal relationship between the d-axis (*I_d_*) and q-axis (*I_q_*) currents that maximized the torque output while ensuring the stator current (*I_s_*) did not exceed the maximum allowable value.

The objective function *F* with equalities constraints is given as follows:

(11) $$Tmax\;=\frac{3}{2}\cdot p\cdot (\lambda_{pm}\cdot I_{qopt}+(L_{d}-L_{q})\cdot {I_{qopt}I}_{dopt})$$

Constraints

(12) $$I_{s}=\sqrt{I_{d\;}^{2}+I_{q\;}^{2}}\leq I_{s\;max}$$

where $I_{s}$ is the motor current and $I_{s\;max}$ is the maximum motor current.

CMA-ES steps:

- Initialize: set initial mean vector (*m*), step size (*σ*), and covariance matrix (*C*);
- Sampling: generate a population of candidate solutions (*x_K_*) using the multivariate normal distribution;

(13) $$x_{K}\sim N(m,\;\sigma^{2}\cdot C)$$

- Evaluation: Evaluate the fitness of each candidate solution using the objectiv function;
- Selection: Select the top-performing candidates based on their fitness values;
- Update: Adapt the mean vector (*m*), step size (*σ*), and covariance matrix (*C*) based on the selected candidates as follows,

(14) $$m=\frac{1}{\tau }\sum_{i=1}^{\tau }x_{i:\eta }$$

where τ is the number of selected candidates, and $x_{i:\eta }$ does fitness sort the selected candidates. The covariance matrix is updated to capture the distribution of successful candidates, promoting better convergence towards the optimal solution.

- Iteration: Repeat the sampling, evaluation, selection, and update steps until convergence or a stopping criterion is met, such as a maximum number of iterations or sufficient convergence of the mean vector.

**Figure 5:**
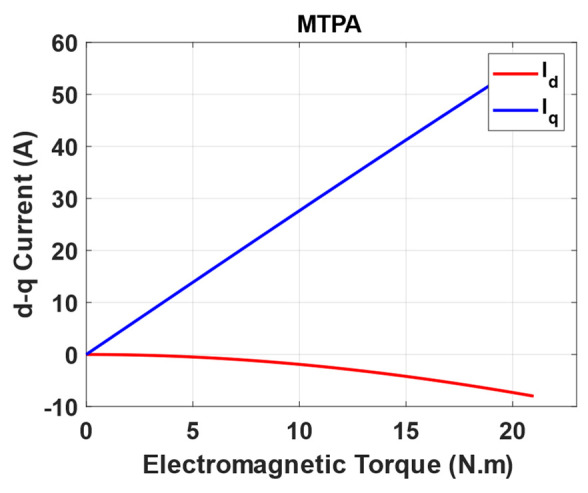
CMA-ES optimization results.

Figure 5 shows the optimization results of the d-q current with their corresponding torque points for MTPA.

**Figure 6:**
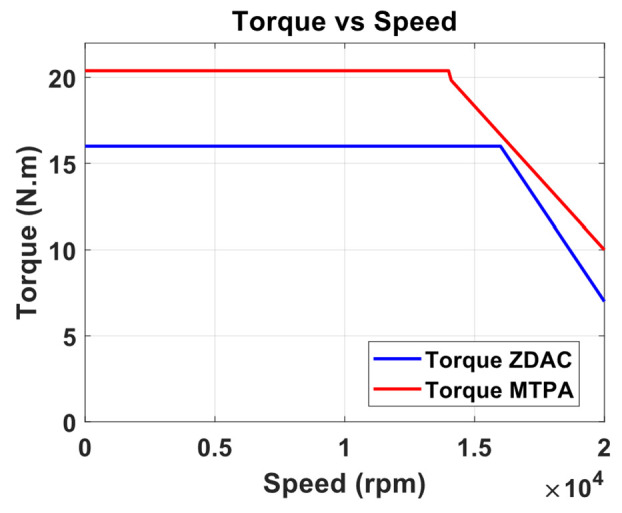
Torque vs. speed from optimal MTPA and ZDAC.

**Figure 7:**
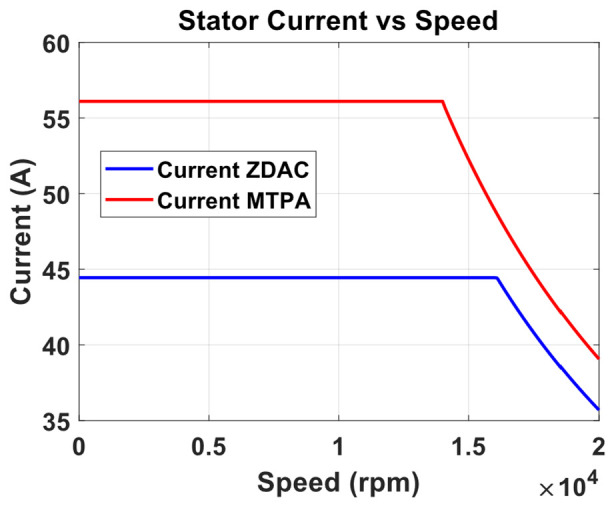
Stator current vs. speed from optimal MTPA and ZDAC.

**Figure 8:**
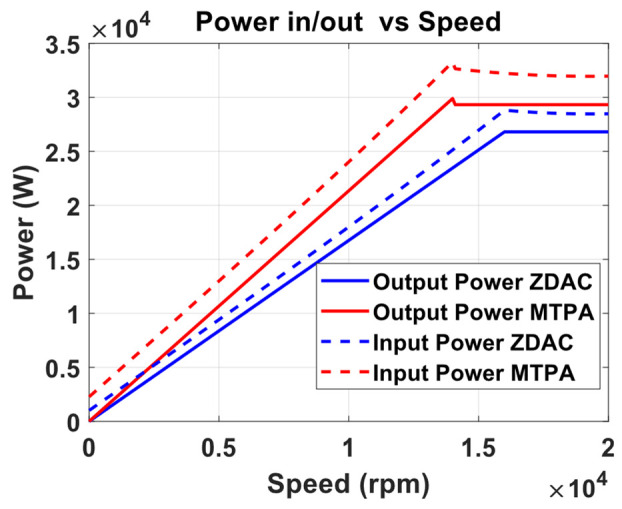
Input/output power vs. speed from optimal MTPA and ZDAC.

**Figure 9:**
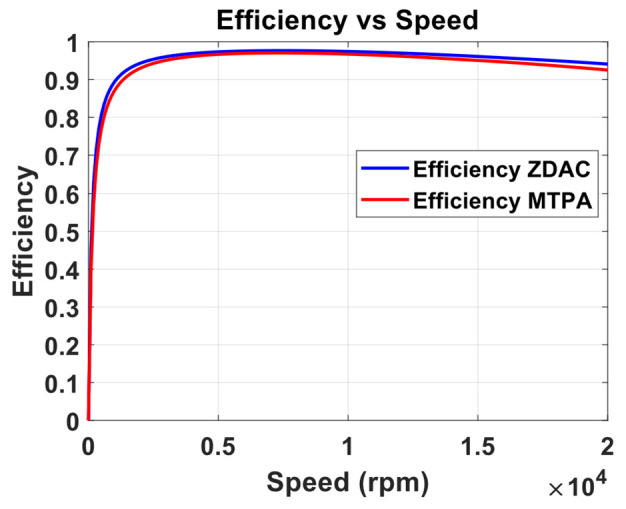
Efficiency vs. speed from optimal MTPA and ZDAC.

A preliminary steady-state analysis was conducted based on the simulation model to compare the performance characteristics under conventional control with ZDAC and optimal MTPA control strategy. The analysis calculated key performance metrics, including torque, current, power output, power input, and efficiency, as shown in the following Figure 6, Figure 7, Figure 8 and Figure 9.

The above figures demonstrate a clear improvement in motor performance under the proposed optimal control strategy. As shown in Figure 6, the ZDAC strategy maintains constant torque up to a base speed of 16,000 rpm, after which the torque declines due to flux weakening in the CP region. In comparison, the optimal control strategy achieves notably higher torque in the CT region. This improvement stems from the application of a negative Id component, which generates additional reluctance torque and allows for more effective utilization of the motor’s magnetic and geometric characteristics. While this strategy results in a slight reduction of base speed to 14,000 rpm—thereby shortening the CT region and expanding the CP region—it does not compromise performance. The torque delivered at and beyond the previous base speed is higher than that with the ZDAC strategy. This shift reflects a deliberate performance-oriented optimization: increasing torque at higher speeds where it is most critical for acceleration, particularly in FEV applications. Figure 7 shows a corresponding increase in stator current due to the applied negative Id, but the current remains within the motor’s rated capacity. Figure 8 illustrates gains in both input and output power, signifying enhanced energy conversion and torque output. Although this results in increased power demand from the battery, such a trade-off is justified in racing scenarios, where acceleration and lap-time reduction take precedence over energy efficiency. Lastly, Figure 9 shows a marginal decline in overall motor efficiency under the optimal strategy. However, this slight loss is outweighed by the significant performance benefits, including higher torque and improved power delivery, which make the proposed control strategy highly advantageous for high-performance electric drive applications.

## 5. Experimental Validation

### 5.1. Testbench

To evaluate the performance of the FEV motor drive, a comprehensive test bench was prepared in a laboratory setting. The test bench was designed to replicate real-world conditions and to allow precise control and measurement of various parameters. The setup includes two coupled PMSMs in a dynamometer (dyno) configuration with a torque sensor in between. It is powered by a battery emulator from Cinergia that accurately mimics the performance of an FEV battery. A heavy-duty traction inverter is used to drive the motor. The inverter is semi-open source and can configure modifications to the control strategy. The whole setup is cooled through a water-cooling system.

**Figure 10:**
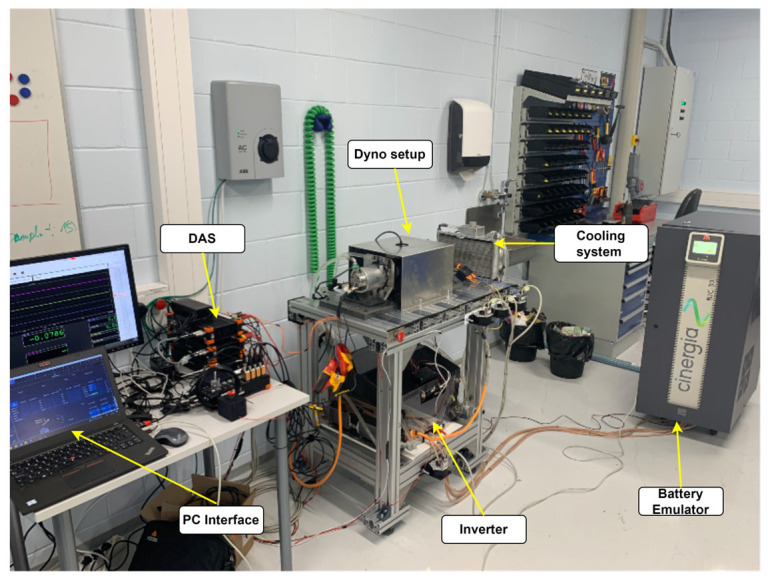
FEV-PMSM drive system testbench.

The DEWESOFT power analyzer and data acquisition system acquire data from sensors (voltage, current, speed, temperature, torque). The test bench was equipped with a computer-based control system to monitor and control the entire setup, enabling the implementation of various test scenarios and real-time data collection. Figure 10 shows the FEV powertrain test bench.

### 5.2. Performance Evaluation

Experimental tests were conducted in the lab to validate the proposed optimal control strategy. The torque-speed profile from the steady-state analysis was used as a reference for the tests. According to Table 2, the following test cases were chosen because they were located within the CT and CP regions of the motor under both conventional ZDAC and optimal control strategies. All tests were performed under controlled thermal conditions to ensure the consistency and reliability of results. During testing, the ambient temperature was maintained at 25 °C, while the motor and inverter temperatures were stabilized at approximately 40 °C by the cooling system.

Initially, the motor drive system was tested under the ZDAC control strategy to establish a baseline performance profile. The proposed optimal MTPA strategy was then implemented into the inverter setting as a look-up table defining direct axis current (Id) as a percentage of quadrature axis current (Iq). The motor drive system was subsequently tested under the proposed optimal control strategy at the same test points. At each operating point on the torque-speed profile, the motor was subjected to the maximum possible load torque corresponding to the point’s location on the profile. This approach ensured a thorough evaluation of the motor’s performance under varying load conditions, reflecting the FEV driving scenario. Appendix A presents the experimental analysis of the motor under conventional ZDAC and optimal control strategies. The comparison included the motor’s electromechanical torque produced, rotor speed, and motor current as the main comparison features. The results highlighted in Table 3 validated the simulation analysis, demonstrating significant improvements in motor performance with optimal strategy.

The above Table 3 summarizes the performance improvements observed in the experimental tests, with the optimal control strategy consistently producing higher torque across all test cases compared to the ZDAC strategy. For Test Case A at 2000 rpm, the MTPA strategy improved torque by 27.53%, demonstrating a substantial enhancement in low-speed performance, which is critical for initial acceleration phases. At 6000 rpm in Test Case B, an 18.21% improvement in torque was observed, indicating effective performance gains at mid-range speeds, essential for maintaining agility and responsiveness. For Test Case C at 10,000 rpm, the torque increase of 24.31% underlines significant gains in higher mid-range speeds, beneficial for steady, high-speed racing segments. Test Case D at 14,000 rpm recorded a 27.04% improvement, showcasing the MTPA strategy’s capability to deliver enhanced performance near the upper limit of the CT region. At 16,000 rpm in Test Case E, the 8.13% improvement, while modest, still indicates better utilization of the motor’s capabilities as it transitions towards the CP region. Test Case F at 18,000 rpm highlighted a 28.07% improvement, demonstrating the effectiveness of the MTPA strategy in extending the motor’s performance envelope further into the high-speed range. Finally, Test Case G at 20,000 rpm showed a 20.41% improvement, indicating that the MTPA control strategy maintained significant performance advantages even at the extreme ends of the motor’s operational range.

On average, the overall improvement ratio in motor performance with the optimal strategy compared to the ZDAC strategy was approximately 22%. These results validate the effectiveness of the MTPA optimization, confirming that the motor drive system achieved higher torque and improved performance under the optimal control strategy. The experimental data closely matched the simulation results as well as steady-state analysis, demonstrating the accuracy of the simulation model and the robustness of the proposed optimal control strategy. The significant torque improvements, especially at higher speeds, indicate the enhanced capability of the motor drive system to meet the demanding performance requirements of FEVs.

However, while the performance of the motor’s torque production is enhanced, the motor’s current within the motor current limit increases. This increases power consumption, leading to a decrease in battery time. Nevertheless, this is not considered a drawback, as in this application, performance is prioritized overpower consumption.

## 6. Conclusions

In this study, the focus was on optimizing the control strategy for high-speed PMSMs used in FEVs. The objective was to enhance the motor’s performance by maximizing torque output while ensuring operational efficiency and stability across the entire speed range. A detailed mathematical model of the PMSM was derived, which served as the foundation for the analysis. Using MATLAB/Simulink, a simulation model of the motor drive system was constructed. The proposed control strategy integrated the principles of maximum torque per ampere with the flux weakening technique, replacing the conventional ZDAC strategy. This combined approach was designed to improve the motor’s performance under high-speed conditions. To determine the optimal d-q axis current ratio for achieving maximum torque without overdesign issues, the covariance matrix adaptation evolution strategy (CMA-ES) was employed. This advanced optimization algorithm effectively identified the optimal current settings that maximized torque output while adhering to the motor’s current limitations. The proposed control strategy was rigorously tested in both simulation and real-world experimental setups. The torque-speed profile from the steady-state analysis was used as a reference for experimental tests conducted on a test bench. The results from these tests confirmed the simulation analysis, demonstrating significant improvements in motor performance with the proposed strategy. The proposed optimal control strategy consistently produced higher torque across all experimental test cases compared to the ZDAC strategy, with improvements ranging from 8.13% to 28.07%. On average, an overall improvement in motor performance of approximately 22% was achieved. These findings confirm the effectiveness of the control strategy optimization in achieving higher torque and improved performance in high-speed PMSMs for FEVs. Significant torque improvements, especially at higher speeds, demonstrated the motor drive system’s enhanced capability to meet the demanding performance requirements of FEVs.

## Figures and Tables

**Table 1:** Understudy FEV-PMSM parameters.

Parameter	Description	Value	Unit
p	Number of pole pairs	5	-
R_s_	Stator resistance	0.10087	Ω
R_c_	Core resistance	27	Ω
L_q_	q-axis inductance	2.4 × 10^−4^	H
L_d_	d-axis inductance	1.2 × 10^−4^	H
P_max_	Maximum output power	35	kW
λ_pm_	Permanent magnet linkage flux	0.048	Wb
N_max_	Maximum speed	20,000	rpm
T_r_	Rated torque	16	N.m
T_max_	Max torque	21	N.m

**Table 2:** Experimental test cases.

Test Case	Reference Speed (rpm)
A	2000
B	6000
C	10,000
D	14,000
E	16,000
F	18,000
G	20,000

**Table 3:** Performance improvement comparison.

Test Case	Average Torque (N·m) ZDAC Strategy	Average Torque (N·m) Optimal Control Strategy	Improvement (%)
A	16.66	21.25	27.53
B	15.7	18.55	18.21
C	16	19.89	24.31
D	15.9	20.2	27.04
E	16	17.3	8.13
F	5.7	7.3	28.07
G	4.9	5.9	20.41

**Figure A1:**
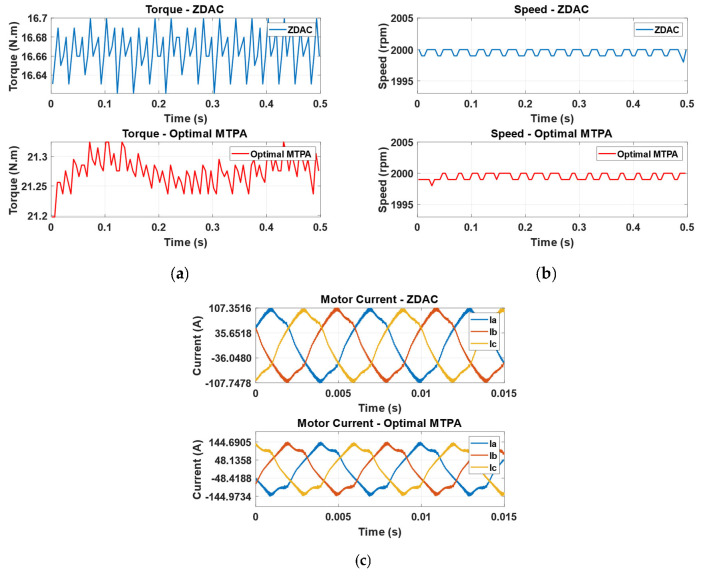
Case A—2000 rpm reference speed.

**Figure A2:**
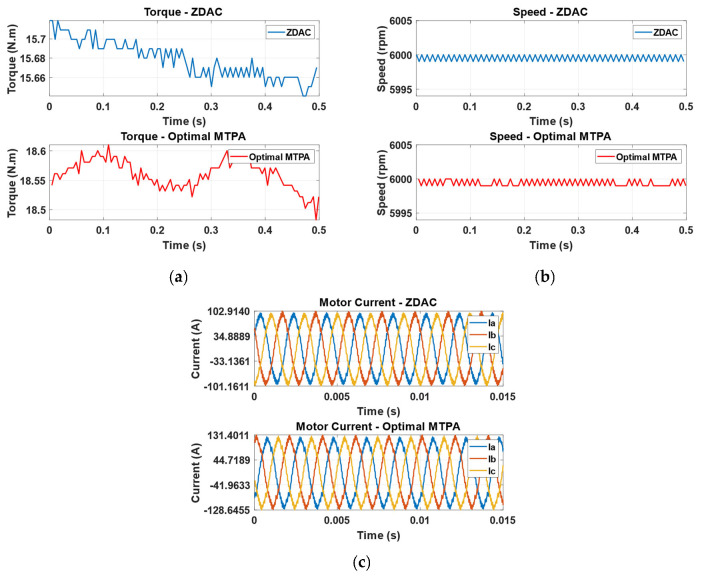
Case B—6000 rpm reference speed.

**Figure A3:**
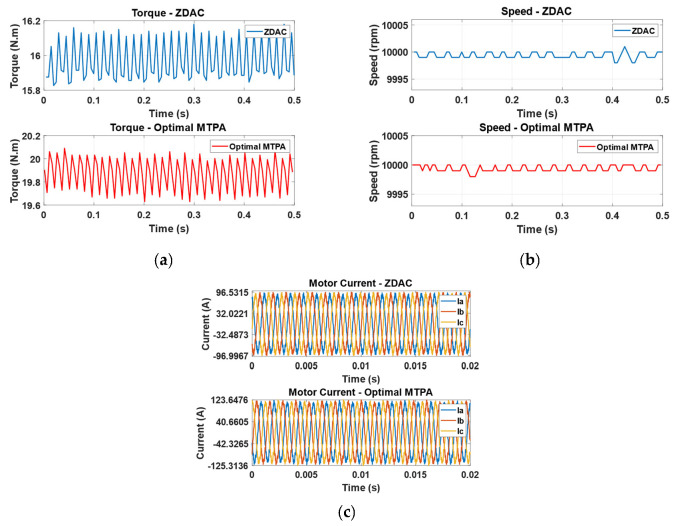
Case C—10,000 rpm reference speed.

**Figure A4:**
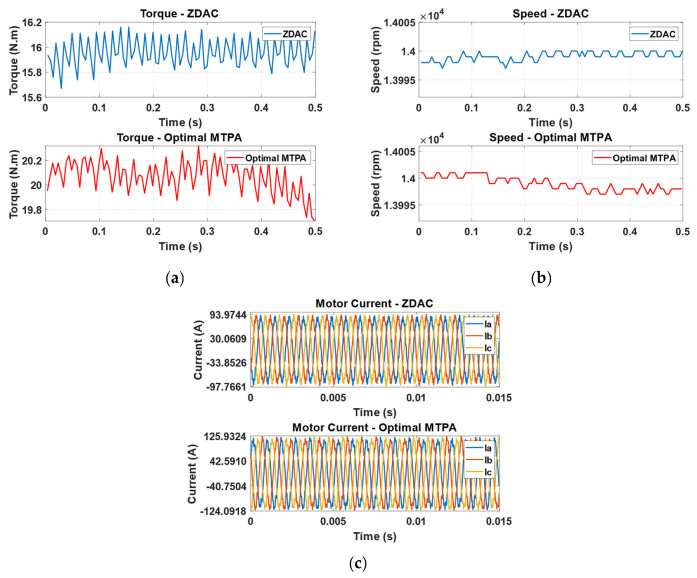
Case D—14,000 rpm reference speed.

**Figure A5:**
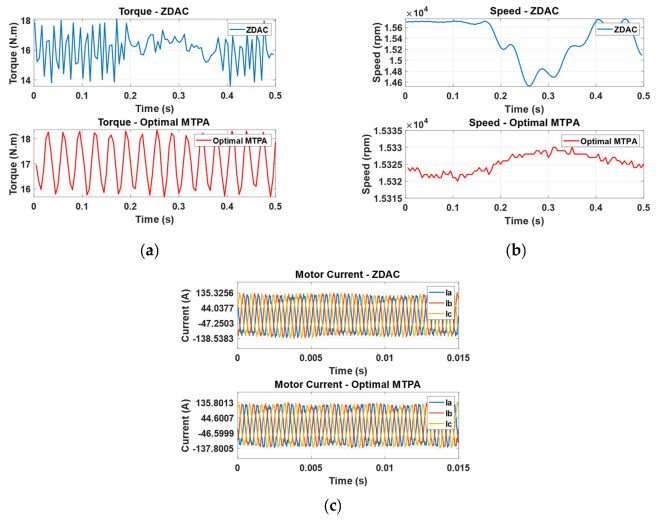
Case E—16,000 rpm reference speed.

**Figure A6:**
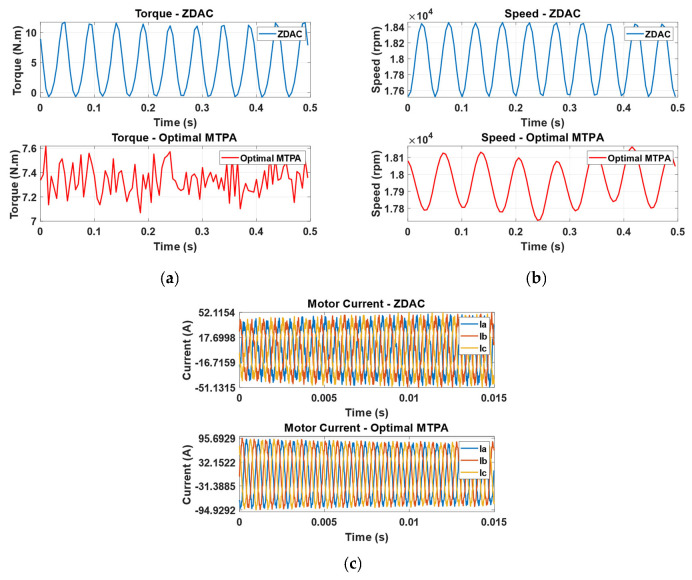
Case F—18,000 rpm reference speed.

**Figure A7:**
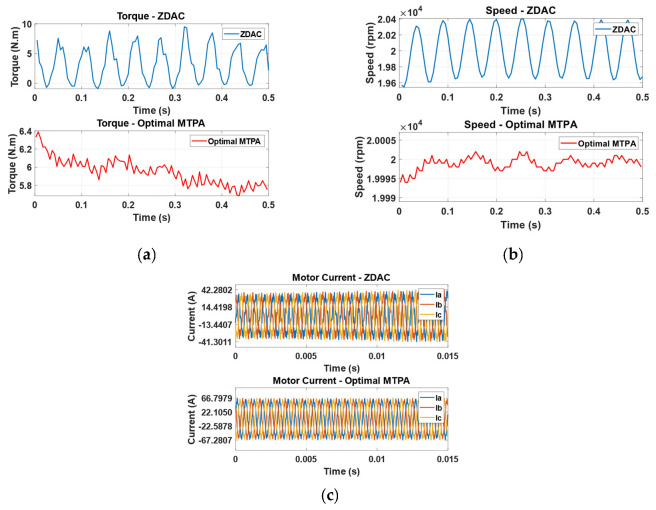
Case G—20,000 rpm reference speed.

## Data Availability

Data are contained within the article.

## Appendix A

Figure A1, Figure A2, Figure A3, Figure A4, Figure A5, Figure A6 and Figure A7 show a comparative analysis of motor performance under conventional ZDAC and proposed optimal MTPA control strategies. Each figure includes three subfigures (a, b, and c), which compare the motor output torque, motor shaft speed, and motor input current, respectively.
